# *Metapogonia snizeki* sp. nov. and a previously unknown female of *M. elgonensis* (Burgeon, 1945) (Coleoptera, Scarabaeidae, Melolonthinae, Diplotaxini)

**DOI:** 10.3897/zookeys.963.54714

**Published:** 2020-08-24

**Authors:** Aleš Bezděk

**Affiliations:** 1 Biology Centre of the Czech Academy of Sciences, Institute of Entomology, Branišovská 31, CZ-370 05 České Budějovice, Czech Republic Institute of Entomology, Czech Academy of Sciences České Budějovice Czech Republic

**Keywords:** Mount Elgon, new species, Tanzania, Uganda

## Abstract

*Metapogonia snizeki***sp. nov.** from northeastern Tanzania is described. The new species is compared with the morphologically closely similar species *M. elgonensis* (Burgeon, 1945). A previously unknown female of *M. elgonensis* is described, and the species is recorded from Uganda for the first time. Relevant diagnostic characters (parameres, shape of male protarsomeres, female pygidium) are illustrated.

## Introduction

The Afrotropical diplotaxine genus *Metapogonia* Lacroix, 2008 (replacement name for *Metagonia* Kolbe, 1899) currently comprises 10 species from northwestern Africa (Senegal, Gambia) to southernmost Africa (Bezděk 2004). Nearly all of these species are known from primary descriptions only, with no comprehensive revisions and faunistic studies available. The members of *Metapogonia* differ from the species-rich and widely distributed genus *Apogonia* Kirby, 1819 mainly in the shape of the male genitalia. Parameres of *Apogonia* are often complex, asymmetrical, and only in some rare cases rather simple and laterally flattened, while those of *Metapogonia* are symmetrical and dorsoventrally flattened. This shape of the male genitalia is shared with the closely related Afrotropical genus *Dichecephala* Brenske, 1895. The main difference between *Metapogonia* and *Dichecephala* is in the shape of the clypeus. In both sexes of *Metapogonia*, the clypeus is simply rounded and slightly emarginated in the middle. The clypeus of *Dichecephala* is, however, strongly sexually dimorphic. The male possesses a clypeus triangularly produced anteriad, the apex of which is deeply emarginate and often bent upward. The clypeus of the *Dichecephala* female is less prominent and always displays two more or less visibly blunt teeth (but never broadly rounded as in *Metapogonia*).

The history of the generic nomenclature of this group of chafers is rather complicated. Kolbe (1899) proposed the genus-group name *Metagonia* as a subgenus of *Apogonia*. Subsequently, Moser (1918) and Burgeon (1945) elevated it to genus rank. This approach was followed by Bezděk (2004), who catalogued all Old World diplotaxine chafers. Unfortunately, all of these authors overlooked the fact that the genus-group name *Metagonia* Kolbe, 1899 was preoccupied by *Metagonia* Simon, 1893 (Aranae, Pholcidae). Nearly simultaneously, Lacroix (2008) and Özdikmen and Demir (2008) proposed replacement names for *Metagonia* Kolbe, 1899. *Metapogonia* Lacroix, 2008 was published on 4 April 2008, while *Bezdekia* Özdikmen & Demir, 2008 on 24 June 2008. Thus, based on the principle of priority, *Metapogonia* is the valid name for this group of chafers (see also Lacroix and Bezděk 2009 for a detailed discussion).

Studies of recently collected material of Diplotaxini from the northeastern part of the Afrotropical Region has revealed a new distinct species of *Metapogonia* as well as additional specimens of both sexes of *M. elgonensis*, a species previously known from the holotype male only.

## Material and methods

A total of 139 specimens were studied. Specimens were examined with an Olympus SZX9 stereomicroscope; measurements were taken with an ocular grid. The habitus photographs were taken using a Canon MP-E 65mm/2.8 1–5× Macro attached to a Canon EOS 550D camera. Partially focussed images of each specimen were combined using Helicon Focus 3.20.2 Pro software. Specimens of the newly described species are provided with one printed red label: “*Metapogonia snizeki* sp. n. | holotypus [or paratypus with type number], sex symbol | Aleš Bezděk det. 2018”. Exact label data are cited for the type material examined. Separate labels are indicated by a double vertical bar “||”, lines within each label are separated by a single vertical bar “|”. Information in quotation marks indicates the original spelling. My remarks and additional comments are placed in brackets, [p] – preceding data (within quotation marks) are printed; [hw] – the same but handwritten. HT – holotype, PT – paratype. The map was composed using SimpleMappr (Shorthouse 2010).

The following codes identify the collections housing the material examined:

**BMNH**The Natural History Museum, London, United Kingdom (Maxwell Barclay, Michael Geiser);

**IECA**Biology Centre CAS, Institute of Entomology, České Budějovice, Czech Republic (Aleš Bezděk);

**ISNB**Institut Royal des Sciences Naturelles de Belgique, Brussels, Belgium (Alain Drumont);

**MFNB**Museum für Naturkunde, Leibniz-Institut für Evolutions- und Biodiversitätsforschung, Berlin, Germany (Bernd Jäger, Joachim Willers);

**MNHN**Muséum National d’Histoire naturelle, Paris, France (Antoine Mantilleri, Olivier Montreuil);

**MRAC**Musée royal de l’Afrique centrale, Tervuren, Belgium (Alice-Marie Buset, Stéphane Hanot);

**NMPC**National Museum, Prague, Czech Republic (Jiří Hájek).

## Taxonomy

### Metapogonia snizeki sp. nov.

Taxon classificationAnimaliaColeopteraScarabaeidae

5D167FF9-9AB0-58B8-83FD-B800DD69CAAD

http://zoobank.org/5EBA52AC-A557-4722-A7AB-43A881518506

**Figures 1–5.**
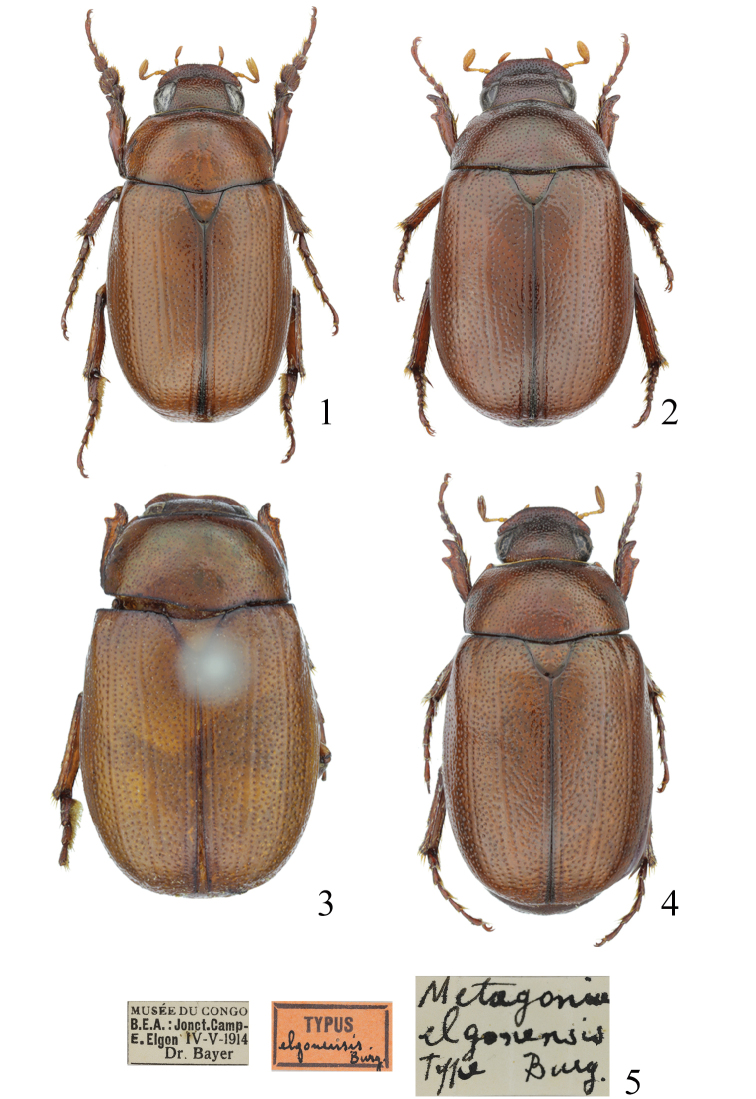
Habitus of *Metapogonia* spp. and associated labels. **1***M. snizeki* sp. nov., HT, 7.9 mm **2***M. snizeki* sp. nov., female PT No. 27, 7.4 mm **3***M. elgonensis* (Burgeon, 1945), HT, 8.1 mm **4***M. elgonensis*, female from Uganda: Kelim River, 7.8 mm **5** labels associated with the holotype of *M. elgonensis*.

**Figures 6–11.**
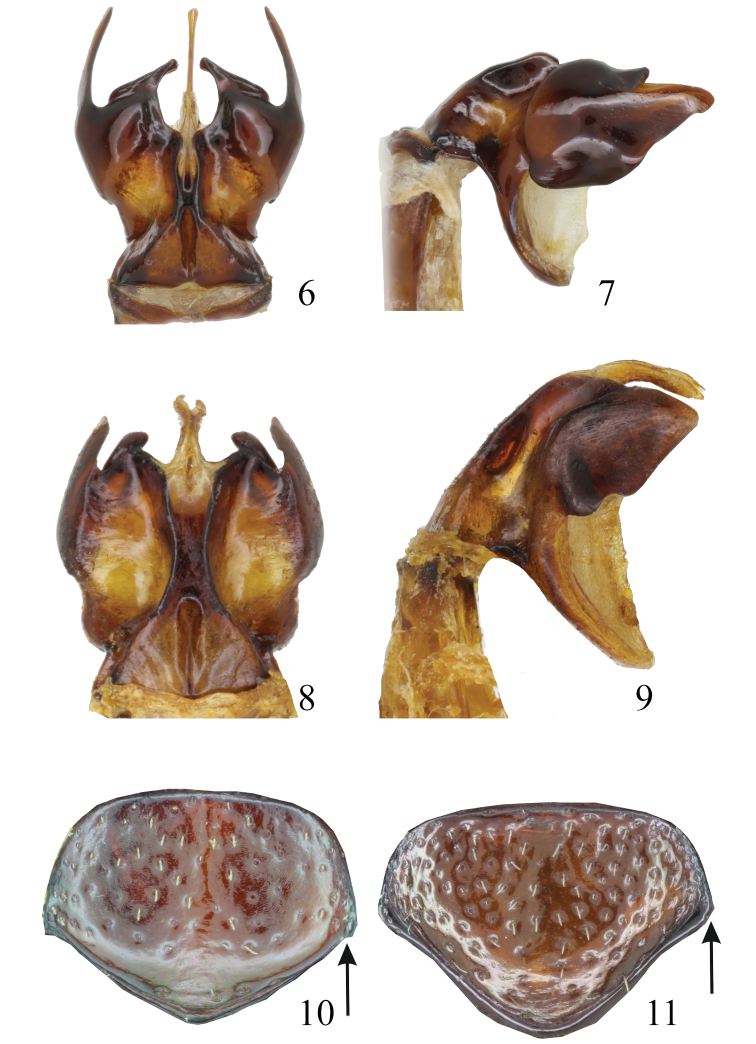
Comparison of diagnostic characters of *Metapogonia* spp. **6***M. snizeki* sp. nov., parameres of HT, dorsal view **7** the same, ventral view **8***M. elgonensis* (Burgeon, 1945), parameres of HT, dorsal view **9** the same, lateral view **10***M. snizeki* sp. nov., female pygidium of PT No. 27 **11***M. elgonensis*, female pygidium of specimen from Uganda: Kelim River. Not to scale.

**Figures 12, 13.**
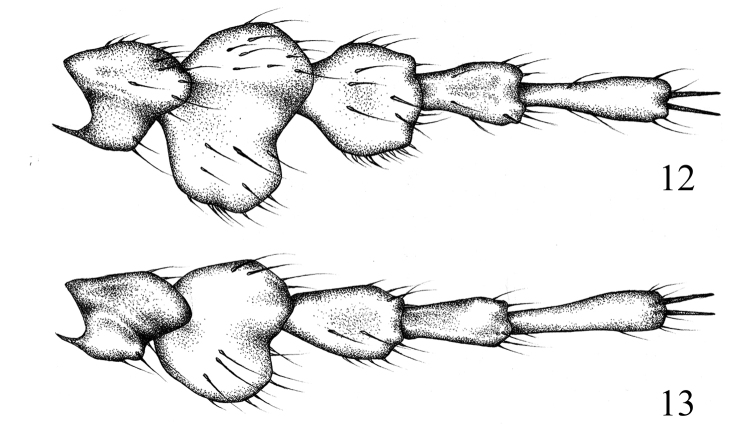
Male protarsomeres of *Metapogonia* spp., dorsal view. **12***M. snizeki* sp. nov., HT**13***M. elgonensis* (Burgeon, 1945), male from Uganda: Atari. Not to scale.

Figures 1 , 2 , 6 , 7 , 10 , 12

#### Type locality.

NEE Tanzania, SSW of Pangani, environs of Pande.

#### Type material.

HT, male, labelled: “Tanzania NEE | SSW of Pangani | Pande env. | 10.3.2002 | lgt. M. Snížek [p]”; PT Nos. 1–8 (males) and 9–35 (females), same data as holotype; PT Nos. 36–46 (males) and 47–83 (females), same data as holotype, but “coast | Forest [p]”; PT Nos. 84–91 (males) and 92–119 (females): “Tanzania NE | Handeni | Makinda env. | 14.3.2002 | lgt. M. Snížek [p]”; PT Nos. 120–121 (females): “Tanzania NE | E of Kiberashi | 15.3.2002 | lgt. M. Snížek [p]”; PT Nos. 122–129 (females): “Tanzania c.or., 350 m | 6°25.4'N, 37°30.4'E | 60 km N of Morogoro | leg. L. Hálková, 13.I.2007 [p]”.

#### Type depositories.

HT and PT no. 1–19, 24–36, 41–84, 87–91, 94–129 in IECA, PT no. 20, 37 in BMNH, PT no. 21, 38 in ISNB, PT no. 22, 39 in MFNB, PT no. 23, 40 in MNHN, PT no. 85, 92 in MRAC, PT no. 86, 93 in NMPC.

#### Description of holotype

**(male).** Body length 7.9 mm. Body elongate, convex, surface brown, moderately shiny, anterior and basal margins of pronotum and sutura narrowly darkened (Fig. 1). Antennae and palpi yellowish brown. Head (except for a few setae on eye-canthus), pronotum and elytra bare, epipleura covered with short but well-visible setae. Legs and ventral surface with sparse, pale setation.

Head. Clypeus transverse, broadly rounded, slightly emarginate in the middle, with coarse and dense punctures. Frons and vertex less densely punctate. Eye canthus prominent, largely fused with clypeus; borderline between eye canthus and clypeus invisible. Eye large, distinctly extended beyond the canthus. Antenna with 10 antennomeres; club trimerous, slightly shorter than antennal shaft. Antennomeres 1–7 with few isolated, erect setae; club sparsely covered with moderately long, erect setae. Labrum transverse, narrow, completely covered by clypeus, thus not visible from above, with coarse irregular punctures bearing moderately long, erect setae.

Pronotum transverse, convex, widest at about the middle, base broader than anterior margin. Anterior angles prominent, acute-angulate; posterior angles obtuse. Anterior margin with membranous border; anterior marginal line incomplete, interrupted in the middle. Lateral marginal line complete; basal marginal line absent. Punctation coarse, punctures separated by 0.5 or less of their diameter, but never confluent. Scutellum triangulate, approximately as wide as long; apex broadly rounded, sparsely punctate in basal half, nearly impunctate apically, completely bare.

Elytron convex, widest about at middle; sutural angle obtuse-angulate. Surface of elytron covered with coarse, irregular punctures. Basal half of epipleuron with a row of short, recumbent setae. Apical half of lateral margin of elytron with membranous border. Macropterous.

Protibia bidentate, basal teeth in some specimens subobsolete; terminal calcar present. Mesotibia and metatibia slightly expanded apically, covered with semirecumbent setae, at about the middle with short, incomplete, transversal carina armed with 2 or 3 short, thick setae. Terminal calcars of mesotibia and metatibia flattened, nearly blunt apically; upper calcar about 1.2 times as long as lower calcar. Protarsomeres 1–4 considerably dilated (Fig. 12); mesotarsomeres and metatarsomeres 1–4 slightly dilated. Tarsomeres 1–4 on all legs with remarkably shortly and densely macrosetaceous pads ventrally. Tarsomere 5 elongate, ventrally and dorsally with few isolated setae. Claws equal, cleft at the apex.

Ventral surface of thorax densely covered with setiferous punctures, setae short, recumbent. Abdominal sternites 3–7 covered with irregular punctures bearing short recumbent or semirecubent setae, setae becoming denser laterally. Abdominal sternites 6 and 7 distinctly narrowed at midline. Abdominal sternite 8 nearly completely retracted beneath abdominal sternite 7, bare, only apical margin with row of erect setae. Propygidium (= abdominal tergite 7) and abdominal sternite 7 completely fused. Pygidium extremely large, convex, irregularly coarsely punctate, apically covered with moderately long, semirecumbent setae, except of smooth depressed midline. Apical and lateral margins of pygidium distinctly bordered.

Male genitalia. Parameres symmetrical (Figs 6, 7), complex, bare, fused basally.

#### Variability.

Male paratypes slightly differs in size (total body length 6.8–8.0 mm, 27 specimens measured), some of them are slightly darker than holotype.

#### Sexual dimorphism.

Female differs from male in the following characters: body length 6.8–7.8 mm (102 specimens measured); antennal club shorter, as long as 6 antecedent antennomeres. Tarsomeres without patches of macrosetae ventrally; pygidium less prominent, nearly flat, with distinct tooth in the centre of lateral margin (Fig. 10).

#### Differential diagnosis.

**Figures 14–16.**
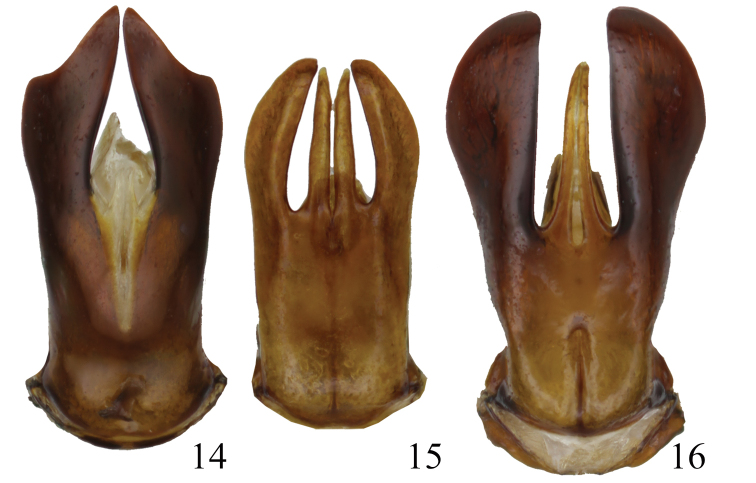
Parameres of *Metapogonia* spp., dorsal view. **14***M. kaszabi* (Frey, 1974) **15***M. parvula* (Moser, 1918) **16***M. platypus* (Kolbe, 1899). Not to scale.

*Metapogonia snizeki* sp. nov. and *M. elgonensis* are the only *Metapogonia* species with deeply bilobed parameres (Figs 6, 8). All other *Metapogonia* species share parameres that are more simply shaped (see e.g. Figs 14–16). The males of *M. snizeki* sp. nov. differ from those of *M. elgonensis* in the shape of the genitalia (compare Figs 6, 7 and 8, 9) and by the shape of protarsomeres, which are more dilated in *M. snizeki* sp. nov. (Figs 12, 13). The females of these two species are very difficult to differentate from the dorsal view, the best identification character seeming to be the shape of the pygidium. The tooth of the pygidium is located nearly in the middle of its lateral margin in *M. snizeki* sp. nov. (Fig. 10), but distinctly more basally in *M. elgonensis* (Fig. 11).

There are three more *Metapogonia* species currently known from Tanzania: *M. kaszabi* (Frey, 1974), *M. parvula* (Moser, 1918), and *M. platypus* (Kolbe, 1899). They are easily distinguishable from *M. snizeki* sp. nov. by the shape of the male genitalia (Figs 14–16) and smaller overall body size (length up to 6.2 mm).

#### Collecting events.

The majority of type material was captured when attracted to light (M. Snížek pers. comm.).

#### Etymology.

The species is named after Miroslav Snížek (Homole near České Budějovice, Czech Republic), one of the collectors of the new species.

#### Distribution.

**Figure 17.**
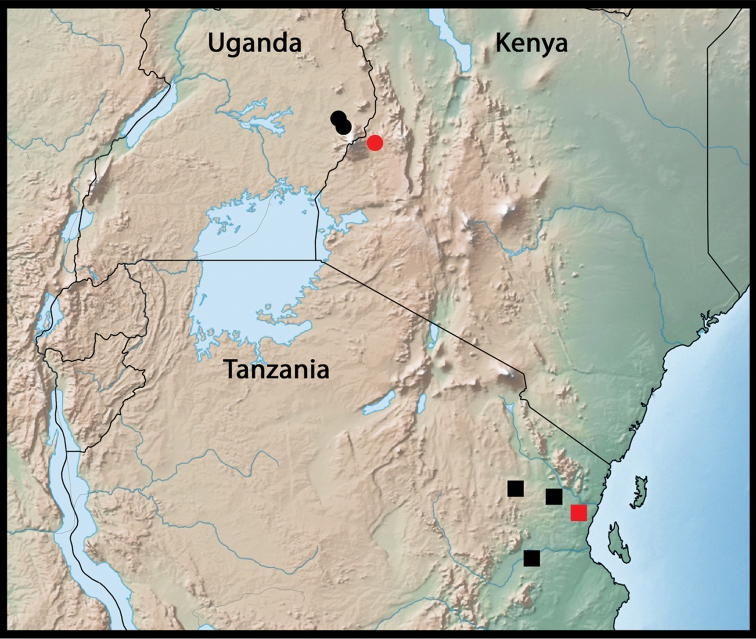
Geographic distribution of *Metapogonia snizeki* sp. nov. (squares) and *M. elgonensis* (Burgeon, 1945) (circles). Red symbols indicate type localities.

Northeastern Tanzania (Fig. 17).

### Metapogonia elgonensis

Taxon classificationAnimaliaColeopteraScarabaeidae

(Burgeon, 1945)

14304A95-DE26-59EC-8E60-043E9B79F9AC

Figures 3–5 , 8 , 9 , 11 , 13

Metagonia elgonensis Burgeon 1945: 17, fig. 19 (primary description); Bezděk 2004: 66 (catalogue).
Metapogonia elgonensis : Lacroix 2008: 69 (new generic combination); Lacroix 2010: 78 (catalogue).

#### Type locality.

“Jonction Camp E. Elgon”.

#### Type material examined.

HT, male, fixed by original designation, 8.1 mm: “MUSÉE DU CONGO | B.E.A.: Jonct. Camp - | E. Elgon, IV-V-1914 | Dr. Bayer [p] || Metagonia | elgonensis | Type Burg. [hw] || TYPUS [p] | elgonensis Burg. [h, red label, black frame] || Metagonia elgonensis | Burgeon, 1945 | holotypus, ♂ | det. A. Bezděk, 2017 [p, red label]”. Deposited in MRAC.

#### Additional material examined

(8 specimens). UGANDA • 1 male and 6 females (IECA), Kelim River, 17.IV.1976, leg. I. Hájek • 1 male (IECA), Kapchorwa district, 5 km NE of Atari, 1066 m a.s.l., 1.47633N, 34.42011E, 26.I.2016, leg. W. & M. Grosser.

#### Diagnosis of females.

Body length 6.9–8.5 mm (6 specimens measured). Body elongate (Fig. 4), convex, surface brown, moderately shiny, anterior and basal margins of pronotum and sutura very narrowly darkened. Antennae and palpi somewhat paler. Head, pronotum, and elytra bare. Legs and ventral surface with sparse, pale setation. Clypeus transverse, broadly rounded, with coarse and dense punctures. Eye canthus prominent, largely fused with clypeus. Eye large, distinctly extended beyond the canthus. Antenna with 10 antennomeres; club trimerous, shorter than antennal shaft. Labrum transverse, narrow, completely covered by clypeus. Pronotum transverse, convex, widest at about the middle. Anterior angles prominent; posterior angles obtuse. Anterior margin with membranous border; lateral marginal line complete; basal marginal line absent. Punctation coarse. Elytron convex, widest about at middle. Surface of elytron covered with coarse, irregular punctures. Macropterous. Protibia bidentate; terminal calcar present. All tarsomeres normally developed. Claws equal, cleft at the apex. Ventral surface of thorax densely covered with setiferous punctures, setae short, recumbent. Pygidium large, almost flat, with distinct tooth in the basal third of lateral margin (Fig. 11).

#### Distribution.

Kenya (Burgeon 1945), first record for Uganda. The species is known from the Mount Elgon area only (Fig. 17).

#### Remark.

The holotype of *M. elgonensis* was collected by Leon Bayer during his 1914 expedition to eastern Africa in the so called “Junction Camp” (Bayer 1923). According to Moreau et al. (1946), the “Junction Camp” was built on a foothill of Mount Elgon in Kenya, the coordinates of the location are approximately 1°7'N, 34°50'E.

The following additional specimens were used for comparison (Figs 14–16):

*Metapogonia kaszabi* (Frey, 1974). Tanzania • 1 male (IECA), Arusha distr., Macumira near Arusha, 1200 m, 15.II.2008, leg. A. Bellmann.

*Metapogonia parvula* (Moser, 1918). Tanzania • 1 male (IECA), Handeni, Makinda env., 14.III.2002, leg. M. Snížek.

*Metapogonia platypus* (Kolbe, 1899). Tanzania • 1 male (IECA), SSW of Pangani, Pande env., 10.III.2002, leg. M. Snížek.

## Supplementary Material

XML Treatment for Metapogonia snizeki

XML Treatment for Metapogonia elgonensis
